# Infantile neuroaxonal dystrophy: Molecular mechanisms and pathogenesis of PLA2G6-associated neurodegeneration

**DOI:** 10.3934/Neuroscience.2025011

**Published:** 2025-05-30

**Authors:** María González-Sánchez, María Jesús Ramírez-Expósito, José Manuel Martínez-Martos

**Affiliations:** Experimental and Clinical Physiopathology Research Group CTS-1039, Department of Health Sciences, School of Health Sciences; University of Jaén, E-23071, Jaén, Spain

**Keywords:** infantile neuroaxonal dystrophy, *PLA2G6*-associated neurodegeneration, neurodegeneration with brain iron accumulation, psychomotor regression, cerebellar atrophy, axonal degeneration, Seitelberger's disease

## Abstract

Infantile neuroaxonal dystrophy (INAD), also known as *PLA2G6*-associated neurodegeneration (PLAN), is a rare, early-onset, autosomal recessively inherited neurodegenerative disease belonging to the group of neurodegenerations with brain iron accumulation (NBIA). The main cause of this disease is bi-allelic mutations in the *PLA2G6* gene, which codes for the enzyme phospholipase A2 type VI. Clinically, it manifests with progressive neurodevelopmental impairment, psychomotor regression, movement disorders, and pyramidal signs. Initially described in the 1950s, the classical form presents in the first two years of life, although later-onset variants are recognized. At the neuropathological level, INAD is characterized by the presence of neuroaxonal spheroids, which are dilations of degenerated axons, located mainly in the white matter, basal ganglia, and cerebellum. INAD is considered a rare or ultra-rare disease, with an estimated prevalence of approximately 1 per million individuals. Diagnosis requires a comprehensive evaluation combining clinical with neuroimaging studies, mainly magnetic resonance imaging (MRI), and genetic analysis. MRI may reveal early cerebellar atrophy and a low-intensity signal in the globus pallidus on iron-sensitive sequences, indicative of iron accumulation. Currently, there is no curative treatment for INAD, so management focuses on providing palliative care and symptom control using a multidisciplinary approach. However, various therapeutic strategies are being investigated, including gene therapy to correct the genetic defect, as well as approaches to modulate pathological pathways such as lipid peroxidation and iron accumulation.

## 1. Introduction

Infantile neuroaxonal dystrophy (INAD) is a rare, early-onset, autosomal recessive neurodegenerative disease included within the group of neurodegenerations associated with brain iron accumulation (NBIA) [1]–[4]. It is clinically characterized by progressive neurodevelopmental impairment, psychomotor regression, movement disorders and pyramidal signs [5]–[7].

Originally described by Seitelberger in the 1950s [3], the classical form of the disease manifests in the first two years of life, although there are phenotypic variants with later onset [1]–[3],[5]–[7]. At the neuropathological level, the disease is defined by the presence of neuroaxonal spheroids, dilated formations of degenerated axons, mainly in the white matter, basal ganglia, and cerebellum [1]–[3],[5],[7]. The main cause of INAD appears to be bi-allelic mutations in the *PLA2G6* gene [8]–[20]. This has allowed it to be reclassified within the spectrum of disorders associated with phospholipase A2 type VI (PLAN) deficiencies [1]–[5],[7],[10],[11],[21].

The present narrative review will attempt to integrate molecular, clinical, and therapeutic advances related to PLAN/INAD pathology. Given that current therapeutic options are limited to symptomatic treatment [2],[3],[5],[22], with management being mainly palliative and focused on symptom control, knowledge of the underlying pathophysiological mechanisms may allow the development of therapies and pharmacological strategies that improve the control of lipid and mitochondrial homeostasis [2],[5],[23]. Since this is a rare or ultra-rare disease, little information is available on its diagnosis and management. The underlying biology is still not precisely understood, and there are no specific treatments. Given this lack of solid scientific evidence, many of the current clinical recommendations are based on expert consensus [24]. This underlines the importance of this review, which compiles and organizes the available knowledge to facilitate knowledge and clinical decision-making.

## 2. Literature search strategy

To support the review, a comprehensive literature search was conducted using PubMed, Web of Science, and EMBASE databases. Articles published between January 2020 and May 2025 were screened using keywords such as “Infantile neuroaxonal dystrophy”, “*PLA2G6*-associated neurodegeneration”, “Neurodegeneration with brain iron accumulation”, “Psychomotor regression”, “Cerebellar atrophy”, “Axonal degeneration”, and “Seitelberger's disease”. Relevant clinical and experimental research and expert guidelines were prioritized to ensure an evidence-based and up-to-date overview.

## 3. General characteristics

Seitelberger's first description was pediatric cases characterized by progressive neurological regression accompanied by specific axonal alterations, visible by light and electron microscopy [3],[20]. These findings consisted of focal dilatations of axons, termed spheroids, representing clusters of disorganized synaptic organelles and vesicles, indicating severe disruption of axonal transport [1]–[3],[5],[7],[14]. In the following decades, this pathology became known as Seitelberger's disease and was classified within the spectrum of neurodegenerative diseases with predominant white matter involvement [3],[8],[25]. Although initially considered an isolated entity, the understanding of this pathology evolved with the development of molecular genetic techniques [2],[10]. These techniques allowed the identification of mutations in the *PLA2G6* gene, which encodes for the enzyme phospholipase A2 type VI, as the main cause of the classical form of the disease [1]–[21]. INAD was thus included in the group of NBIA, more specifically termed as *PLA2G6*-associated neurodegeneration (PLAN) [1]–[11],[21]. However, the term “infantile neuroaxonal dystrophy” is still used to refer to the early-onset form, whereas the term “PLAN” encompasses a broader spectrum of clinical phenotypes related to mutations in the same gene [2],[7]–[9],[14],[17],[20].

**Figure 1.**
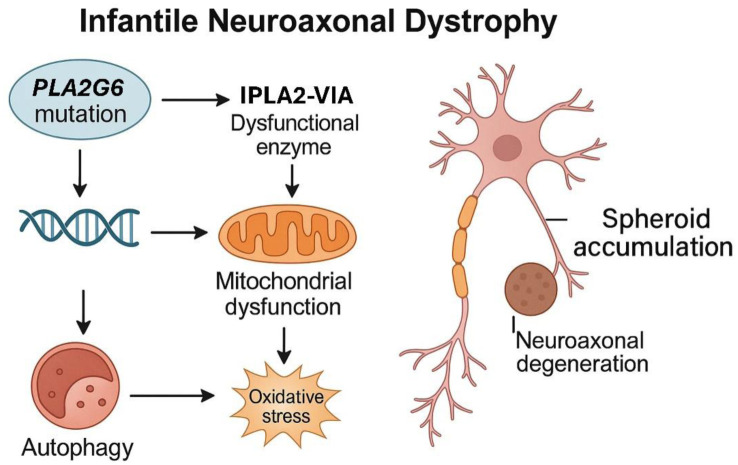
Pathogenic mechanism of infantile neuroaxonal dystrophy. The mutation in the PLA2G6 gene leads to dysfunction of the iPLA2-VIA enzyme, resulting in mitochondrial dysfunction, increased oxidative stress and alterations in autophagy. These processes contribute to spheroid accumulation and neuroaxonal degeneration, which are characteristic of the disease.

From a clinical point of view, INAD is distinguished by its onset in the first two years of life [1]–[8],[17]–[19],[21], a rapid and progressive evolution [1]–[4],[7],[14],[17],[21], the consistent presence of extrapyramidal signs, psychomotor retardation, and cerebellar atrophy on neuroimaging tests [1]–[7],[15],[16],[18]–[21], as well as the characteristic appearance of neuroaxonal spheroids in histological studies [1]–[3],[5],[7],[8],[14],[21] (Figure 1).

An atypical or juvenile form of PLAN, also known as atypical neuroaxonal dystrophy (ANAD), has also been described with a later onset in late childhood or adolescence and a slower progression [1],[3],[26]. Late-onset phenotypes tend to be more heterogeneous. Similarly, autosomal recessive spastic paraparesis, characterized by mutations in *PLA2G6* without visible neurodegeneration, and hereditary spastic paraparesis (HSP) have been described as other consequences of mutations in *PLA2G6*, both involving motor pathway degeneration [1],[3],[4],[6]. Finally, neonatal encephalopathy with epilepsy, a severe perinatal form, is also included in this group. In fact, the evolution in the definition of the disease reflects the way in which knowledge of these pathologies has progressed, from neuropathological descriptions that characterized the presence of axonal spheroids [8] to the current classification based on molecular mechanisms shared by mutations in the *PLA2G6* gene [1],[3],[14].

INAD is no longer considered an isolated clinical entity but as the most severe and earliest-onset form of the spectrum of pathologies associated with *PLA2G6* mutations [1]. Its diagnostic implications are important, as it allows the identification of pathogenic variants in *PLA2G6* [1],[3],[14] and also affects prognosis because the specific mutation, together with the age of onset, is related to disease progression [1]. In a certain way, knowledge of the underlying mechanisms also has therapeutic implications, by suggesting the lines of development of possible interventions [2].

## 4. Epidemiology

Since INAD is a rare or ultrarare neurodegenerative disease, its prevalence is not accurately known due to its low incidence [1],[4],[6],[10],[17],[20],[21], underdiagnosis [1],[4],[6],[10],[17],[20],[21], and the possible lack of comprehensive population-based registries in many countries. Nevertheless, its prevalence has been estimated at approximately 1 per 1,000,000 individuals [2]. While about 150 cases of PLAN have been reported [7], INAD is the most common form in childhood.

Genetic studies indicate that there is a higher incidence in regions with a high rate of inbreeding, as observed in Iran [6] and Pakistan [21]. The high prevalence of consanguineous unions in the latter country, approximately 70%, could contribute significantly to a higher incidence of autosomal recessive diseases, such as INAD [21]. Indeed, in these populations, autosomal recessive mutations in the *PLA2G6* gene are the root cause of this pathology [1]. More than 200 documented pathogenic/likely pathogenic variants have been identified in the ClinVar database for the *PLA2G6* gene [10]. Cases of dystonia-parkinsonism associated with mutations in *PLA2G6* have been reported in Iran [6],[27]. In Europe, it is estimated to affect less than 0.01 persons per 10,000 population, which is equivalent to less than 500 cases in the entire European Union (including Norway, Iceland, and Liechtenstein). This figure places it below the threshold for designation as an ultrarare disease (5 cases per 10,000). In Spain, INAD is an extremely rare disease. According to the most recent data, only 5 children have been diagnosed. Moreover, as most patients die before adolescence due to respiratory complications, it also has a low cumulative prevalence.

In terms of sex distribution, INAD is an autosomal recessive disorder, so it affects males and females equally, as both sexes are equally likely to inherit two altered copies of the *PLA2G6* gene [1],[21]. One study in Pakistan described a family where three males were affected [21], which is consistent with the autosomal recessive inheritance pattern in that specific family. Another study reported two affected male twins [15].

Although the etiology of INAD is mainly focused on genetic mutations [1],[2], differences in clinical presentation between individuals with the same mutations suggest the possible influence of modifying factors on the clinical phenotype, especially in atypical forms of PLAN, which is still under investigation [7].

Certainly, the increase in the number of identified cases of INAD in recent years has been a consequence of the increasing access to next-generation genetic sequencing (NGS) techniques [2],[7],[10],[15]–[17],[20],[21],[28], in addition to the growing medical awareness of early-onset neurodegenerative syndromes [7],[16],[20]. Having these genetic tests available has also facilitated the confirmation of INAD diagnosis with the identification of pathogenic variants in the *PLA2G6* gene, even avoiding invasive biopsies [2],[10],[15],[20],[21]. And although studies with significant numbers of patients remain limited due to the rarity of the disease [1],[2],[9],[10], some international cohorts have been able to be formed [1],[9], which are facilitating the understanding of the natural history of the disease, the development of clinical assessment scales, and genetic and therapeutic research projects, facilitating the design of clinical trials [1],[9]. Organizations such as The INADCure Foundation, based in the United States, promote and fund international projects, including the development of gene therapies in collaboration with the NBIA Research Group at Oregon Health & Science University (OHSU), in Portland, Oregon. This foundation also promotes the creation of international registries and the participation of families and clinical centers worldwide [2]. Other international multicenter studies [1] demonstrate collaborative efforts to address this rare disease.

However, the epidemiology of INAD still has important limitations [3],[4],[7]. Phenotypic heterogeneity, including variation in age of onset and clinical presentation [7],[8],[21], complicates its early identification [3],[4],[6],[7]; on multiple occasions, clinical findings can be confusing, and early signs may not always be specific [4],[6],[7]. In addition, many databases may not clearly differentiate classical and atypical forms of PLAN [7],[8],[21], making it difficult to obtain accurate epidemiological data for each subtype. Similarly, the lack of longitudinal registries limits the assessment of the real impact of the disease in terms of health burden, life expectancy, and social needs [2],[9]. Although the progressive course of the disease is known [1],[2],[7], detailed and age-specific information may be limited due to the retrospective nature of many studies [1]. Although INAD is a rare disease, its clinical and familial impact is enormous [2],[3], and adequate knowledge by medical professionals is crucial for early diagnosis and being able to provide appropriate genetic counseling to affected families [3],[4],[18].

## 5. Genetic and molecular basis

As stated before, INAD is part of the group of diseases associated with mutations in the *PLA2G6* gene [1],[2],[17],[21]. This gene is located in the 22q13.1 region of chromosome 22 [1],[17] and encodes for the enzyme phospholipase A2 type VI, also known as iPLA2-VIA or iPLA2β [1],[2],[5]–[8],[10],[14],[17],[18],[20],[21],[29],[30]. Bi-allelic mutations in this gene are well characterized as being responsible for INAD/NBIA/PLAN [1],[5],[6],[8],[17],[20],[21], whose diagnosis is precisely established by identifying pathogenic bi-allelic variants in *PLA2G6*. The determination of the specific phenotype is based on clinical, neurophysiological, radiographic, and laboratory features [1].

iPLA2-VIA is a cytosolic enzyme that catalyzes the hydrolysis of the ester bond at the sn-2 position of membrane phospholipids [1],[2],[7],[10],[14],[17],[20],[21], generating free fatty acids (such as arachidonic acid) and lysophospholipids [1],[2],[7],[14],[20],[21]. This activity is essential for several key processes. First, it enables the turnover and maintenance of cell membrane integrity [1],[5],[7],[10],[14],[17],[18],[21]. Indeed, loss of iPLA2-VIA function is associated with functional and structural abnormalities, especially in neurons, where damaged membranes accumulate as spheroids, leading to early cell death and neurodegeneration [5]. Second, iPLA2-VIA is also involved in lipid-mediated cell signaling [1],[18],[21]. Phospholipid hydrolysis products serve as precursors for biologically active metabolites involved in cellular signal transduction [21]. Third, iPLA2-VIA participates in the hydrolysis of peroxidized fatty acids, which are formed due to oxidative stress [2]. Dysfunction of iPLA2G6 can also lead to elevated mitochondrial lipid peroxidation [5],[7],[10],[18]. Finally, this activity is involved in balancing calcium homeostasis and mitochondrial function [1],[5],[7],[10],[18], showing that loss of *PLA2G6* can alter mitochondrial Ca^2+^ uptake in neurons [29], also helping to promote lipid peroxidation, mitochondrial membrane abnormalities, and its dysfunction [18].

iPLA2-VIA shows elevated expression in metabolically highly active tissues, as occurs with the brain, spinal cord, muscle, and retina [1],[17],[21]. This explains, at least in part, the selective vulnerability of the central nervous system when its function is compromised [1],[5]. In the human brain, iPLA2-VIA is expressed especially in the substantia nigra, cortex, and hippocampus [6]. In general, loss of iPLA2-VIA enzymatic activity leads to accumulation of damaged membrane phospholipids and failures in multiple essential cellular processes, which ultimately result in axonal degeneration and neurodegeneration characteristic of the disease [1],[5],[7],[18],[21].

To date, more than 100 different pathogenic variants have been described for *PLA2G6*, and more than 200 are classified as pathogenic/likely pathogenic variants [4]. Most are missense mutations, but frameshift variants, splice-site losses, complete or partial gene deletions, large intragenic deletions, and insertion mutations have also been described [28]. The most severe mutations are associated with the classic infantile form of PLAN (i.e., INAD) and lead to a significant decrease in phospholipase activity. Patients with severe INAD carry two null mutations in *PLA2G6*, resulting in the complete absence of protein. On the other hand, hypomorphic mutations (such as some nonsense mutations) that might allow residual enzyme activity may result in atypical forms with later onset, such as ANAD or parkinsonism with adult-onset dystonia [17].

This genotype-phenotype correlation, although still incomplete, has been observed in multiple international cohorts. However, it is important to note that phenotypic variability may exist even among patients sharing the same genotype or belonging to the same family. The phenotypic consequence of these mutations could be related to the combination of heterozygous mutations. Pathogenic mutations in *PLA2G6* have been described in all exons, indicating that disease-causing mutations do not occur at specific critical points in an exon [1],[21].

### 5.1. Other genes involved in similar phenotypes

Although most cases of INAD are associated with *PLA2G6*, there are similar clinical phenotypes caused by mutations in other genes. This is the case with the FA2H gene, associated with spastic leukodystrophy with spheroids [7] that leads to a form of NBIA [7]. Mutations in other genes associated with NBIA are *ATP13A2*, *WDR45*, and *CP*, among others [31], which can present features similar to PLAN. Finally, *C19orf12* causes mitochondrial membrane protein-associated neurodegeneration (MPAN), which can be confused with atypical forms of PLAN [7]. MPAN is considered another subtype of NBIA [32]. This clinical overlap and genetic heterogeneity imply that diagnosis must be based either on gene panels or whole exomes [19]. Indeed, it has been observed that mutations in *PLA2G6* are only detected in approximately 85% of patients with the INAD phenotype [1]. This lack of genotypic confirmation may be due to the fact that gene sequencing may not be inclusive of promoter areas or introns, or that other unrecognized genes may be involved in this phenotype. It has also been suggested that INAD is genetically heterogeneous, as linkage data supported the existence of at least one additional locus for INAD [1].

### 5.2. Experimental models

Mouse animal models with *PLA2G6* deletion reproduce many of the neuropathological alterations observed in humans, such as the appearance of spheroids, progressive neuronal loss, motor deficits, and iron accumulation in brain structures such as the basal ganglia [33]–[35]. Ultrastructural analysis of neurons in *PLA2G6* knockout mice shows mitochondria with branched and tubular ridges or degenerated ridges, a collapsed axonal cytoskeleton, and partial membrane loss at axon terminals [36]. At the microscopic level, these features manifest as axonal swelling and spheroid bodies in presynaptic terminals. These models have also served to investigate underlying pathophysiological mechanisms, such as alterations in lipid homeostasis and mitochondrial dysfunction [2],[36]. Altered ATP-induced calcium signaling in astrocytes, as well as altered mitochondrial calcium uptake in neurons, has also been demonstrated in these murine models [29],[30].

In vitro models have also been developed with human induced pluripotent stem cells (hiPSCs) carrying specific mutations [12]. These cellular models have allowed the evaluation of new therapies aimed at restoring lipid function or improving mitochondrial homeostasis [2]. In addition, in vitro models may be useful to study the pathogenic mechanism underlying INAD at the cellular level [2].

## 6. Pathophysiology

The progressive neurobiological alterations that characterize INAD, mainly at the central nervous system level, as a consequence of the enzymatic dysfunction of iPLA2-VIA encoded by the *PLA2G6* gene, reflect the disruption of multiple important cellular processes that converge in axonal degeneration, synaptic disruption, and neuronal loss, key elements that explain the progressive and multisystemic symptomatology observed in patients.

### 6.1. Axonal degeneration and spheroid formation

The most characteristic neuropathological finding of INAD is the presence of neuroaxonal spheroids [1],[5],[8]. These represent focal dilations of axons and distal nerve terminals, with intracytoplasmic accumulation including swollen mitochondria with electron-dense granules, tubules of variable size, myeloid bodies, focal filamentous aggregates, and membrane-bound electron-dense aggregates and lysosomes, as well as abnormally phosphorylated neurofilaments and ubiquitin [5]. Ultrastructurally, swollen axons show an attenuated myelin sheath and depletion of myelin material [5]. This alteration reflects a disruption of anterograde and retrograde axoplasmic transport, essential for neuronal survival and functionality [5]. Spheroids are notably predominant in the sensory nuclei of the medulla, in the brainstem, and in the dorsal horn and funiculi of all levels of the spinal cord. They are also seen in the periaqueductal gray matter, myelinated fiber tracts, and trochlear nuclei [5]. In the brain, diffuse spongiosis of the cerebral cortex with some neuronal loss and astrogliosis is present, and the basal ganglia (caudate, thalamus, and putamen) show extensive astrogliosis with diffuse axonal swelling. Accumulation of these spheroids in the gracile and cuneate nuclei of the medulla can lead to an increase in clavate size [5],[8],[37].

### 6.2. Mitochondrial and bioenergetic dysfunction

Neurons deficient in iPLA2-VIA also show alterations at the mitochondrial and cellular level, which may lead to a state of hypometabolism and increased vulnerability. Regarding mitochondrial fragmentation, ultrastructural analyses of neurons in *PLA2G6* knockout mice showed mitochondria with branched and tubular ridges, and mitochondria with degenerated ridges [2].

Regarding increased oxidative stress, as previously mentioned, loss of iPLA2-VIA function is associated with elevated lipid peroxidation and mitochondrial dysfunction [2],[5],[10],[30],[38]. Neuronal cells are highly susceptible to oxidative damage due to their high content of unsaturated fatty acids, and accumulation of oxidized phospholipids can lead to lipid peroxidation and generation of reactive oxygen species [10].

A reduced mitochondrial membrane potential has also been demonstrated in situ in neurons from iPLA2 VIA-deficient mice [5],[29],[30]. This reduced mitochondrial potential may account for the decreased rate of Ca^2+^ uptake by mitochondria [30]. The dissipation of mitochondrial membrane potential likely results in less efficient ATP production, leading to a compensatory increase in anaerobic metabolism, especially under conditions of increased energy demand [39]. As a result, these defects can induce a state of neuronal hypometabolism. Mitochondrial dysfunction and impaired phospholipid metabolism can compromise electron transport chain activity and ATP production [10],[40].

Predisposition to apoptosis is also increased due to reduced Ca^2+^ uptake capacity by mitochondria in INAD models. This may lead to increased vulnerability of mitochondria to Ca^2+^ overload during pathological elevations of intracellular Ca^2+^, which may trigger opening of the mitochondrial permeability transition pore and release of apoptotic factors [30].

As a whole, the reduced capacity to handle peak energy demands as a consequence of mitochondrial dysfunction and hypometabolism implies that damaged or less efficient mitochondria cannot adequately respond to the high energy demands of active neurons. Cells of the nervous system with high metabolic demands and exposure to oxidative stressors, such as those of the cerebellum and basal ganglia, are highly sensitive to defects in phospholipase A2 activity [1]. Furthermore, metabolite accumulation and oxidative stress damage are especially notable in key brain areas such as the basal ganglia [2].

### 6.3. Disruption of lipid metabolism

Altered phospholipid homeostasis compromises the structure and function of cell membranes, including synaptic vesicles, myelin, and mitochondrial membranes [2],[5],[10],[14],[18],[21]. This contributes significantly to the neurodegenerative processes observed in INAD and other PLAN [6],[7],[11],[14],[31].

Regarding the instability of synapses, it should be noted that iPLA2-VIA is involved in the remodeling of phospholipid membranes in axons and synapses [3],[5],[7]. Its deficiency results in the accumulation of damaged membranes as spheroids, which affects presynaptic terminals [2],[5],[8],[14]. The accumulation of dysfunctional mitochondria and vesicles in axon terminals, characteristic of INAD [7],[8], suggests impaired neurotransmitter release and reuptake, leading to synaptic instability [7],[10],[21]. In addition, the degeneration of presynaptic membranes observed in *PLA2G6*-deficient animal models [2],[36] also contributes to synaptic dysfunction.

With regard to altered lipid-mediated neuronal signaling, it should be noted that phospholipids and their hydrolysis products act as precursors of multiple signaling molecules involved in intracellular signaling pathways [10],[14]. Dysregulation of phospholipid metabolism can disrupt their production, leading to aberrant cell signaling and impaired neuronal function [10]. This can affect synaptic function, as stated before, but also neurotransmitter balance and neuronal excitability [10]. iPLA2-VIA is also involved in the regulation of store-operated calcium entry (SOCE) into cells [5],[29],[30], a crucial process for cell signaling. Its dysfunction can alter calcium fluxes, affecting neuronal communication and synaptic plasticity [29].

Regarding the decrease in axon regenerative capacity, accumulation of organelles and proteins in distal axons also participates in spheroid formation [8],[21]. This indicates an impairment of axoplasmic transport [5]. Axoplasmic transport is essential for the delivery of components necessary for axonal maintenance and regeneration. Altered lipid composition of axonal membranes, in addition to mitochondrial dysfunction [5],[10], compromises the structural and energetic integrity of the axon, decreasing its ability to regenerate after damage. Loss of iPLA2-VIA function is associated with structural abnormalities in neurons, leading to early cell death and neurodegeneration [5]. In addition, studies in *PLA2G6*-deficient mice show collapse of the axonal cytoskeleton and partial loss of membrane in axon terminals [2], suggesting an intrinsic difficulty in maintaining the axonal structure necessary for regeneration.

### 6.4. Brain iron accumulation

Although iron accumulation is not as prominent in infantile forms of PLAN (INAD) as in other forms of NBIA, it may be detectable by MRI [31]. Hypointense signal has been observed in the globus pallidus on T2 and SWI sequences [21],[31],[41]. In ultrastructural evaluations of swollen axons in patients with *PLA2G6* mutations, the presence of iron deposits has also been observed [5]. Iron accumulation may participate in the generation of reactive oxygen species (ROS), amplifying oxidative damage and promoting neurodegeneration [5]. *PLA2G6* has been suggested to be mutated in neurodegenerative disorders with high brain iron content [42]. The pathology associated with *PLA2G6* mutations, although distinctive in its genetic cause, shares with other neurodegenerative diseases, such as Alzheimer's and Parkinson's, certain key pathogenic mechanisms. These include mitochondrial dysfunction and increased oxidative stress, including lipid peroxidation, to which iron accumulation contributes; disruption of the autophagic-lysosomal system and the ubiquitin-proteasome system, resulting in the accumulation of misfolded proteins and damaged organelles; and neuroinflammation. These underlying processes together contribute to the axonal and neuronal degeneration characteristic of the disease [42].

### 6.5. Alteration of autophagy and protein degradation

Several studies have evidenced a dysfunction of the autophagic-lysosomal system and the ubiquitin-proteasome system in PLAN models [5]. This dysfunction leads to the accumulation of misfolded proteins and damaged subcellular organelles. In a *PLA2G6*-deficient mouse model, altered membrane homeostasis and accumulation of ubiquitinated proteins were observed [5]. Ultrastructural analyses of neurons in *PLA2G6* knockout mice are consistent with this molecular pathology, showing degenerated mitochondria, axons with cytoskeleton collapse, and partial membrane loss in axonal terminals [33],[36]. Accumulation of damaged organelles and misfolded proteins can trigger cellular stress and programmed cell death pathways [5]. These processes may also contribute to chronic neuroinflammation, as observed at the Purkinje cell level and responsible for cerebellar atrophy [5].

### 6.6. Neuroinflammation

The neuronal dysfunction and progressive cell death observed in PLAN are related to the activation of neuroinflammation in the absence of *PLA2G6* [5],[6],[18]. Thus, in patients with *PLA2G6* mutations, astrogliosis has been described in the cerebral cortex, caudate, thalamus, and putamen [8]. In the mouse model with a deficient *PLA2G6* gene, glial cell activation was observed along with cerebellar atrophy and Purkinje cell loss [5],[18]. In histopathological analysis of patients with INAD, extensive astrogliosis has been found in the cerebral cortex, caudate, and putamen [8].

However, it remains to be defined whether neuroinflammation is a secondary consequence of progressive neuronal death that amplifies damage, demyelination, and clinical deterioration. Although this is a common mechanism in neurodegenerative diseases [5], studies in mice suggest a connection between neuronal dysfunction and glial response.

### 6.7. Selective vulnerability of the CNS

There is selective vulnerability of certain CNS regions in INAD [5],[6],[18]. At the cerebellar level, progressive cerebellar atrophy is a common feature in INAD, responsible for ataxia [1],[7],[15],[17],[19],[21],[43],[44]. MRI typically shows cerebellar atrophy, which may be associated with hyperintensity of the cerebellar cortex on T2-weighted images [7],[15],[19],[21],[43]. Purkinje cell loss and glial cell activation in the cerebellum have also been observed in *PLA2G6*-deficient mouse models [5],[18],[20]. The high metabolic demand and exposure to oxidative stressors typical of nervous system cells, particularly in areas such as the cerebellum, make them highly sensitive to defects in phospholipase A2 activity [1].

Basal ganglia involvement is associated with dystonia and may contribute to symptoms of Parkinsonism [1],[7],[17],[21],[44]. Iron accumulation in the globus pallidus and substantia nigra is an occasional finding on MRI of patients with INAD [7],[19],[20],[21],[43],[44], although it is not always present, especially in the early stages [4]. Mitochondrial dysfunction and lipid peroxidation, consequences of *PLA2G6* loss of function, may contribute to basal ganglia vulnerability and iron accumulation [5].

Spasticity, a manifestation of corticospinal degeneration affecting the motor cortex, is a common neurological finding in INAD [1],[7],[17],[43]. Studies have reported signs of pyramidal pathway involvement [7],[43]. Accumulation of misfolded proteins and damaged subcellular organelles, as well as altered membrane homeostasis and neuroinflammation, may contribute to neuronal degeneration in the motor cortex and corticospinal pathways [5].

Optic nerve and retinal involvement explain the early visual loss seen in many patients with INAD, which can manifest as optic atrophy and abnormalities in visual evoked potentials (VEP) [1],[7],[17],[19],[20],[21],[43],[44]. Nystagmus and strabismus are also frequent ophthalmologic findings. Axonal dysfunction and spheroid accumulation also affect peripheral nerves, suggesting widespread vulnerability of neurons with long axons, including retinal ganglion cells and their axons in the optic nerve [1],[7].

## 7. Clinical manifestations

INAD manifests as a rare, early-onset, progressive, and disabling neurodegenerative disorder affecting multiple neurological domains. The first symptoms usually appear between 6 months and 3 years of age [8]–[10],[13], with a typical onset around 12 months, after an initial period of apparently normal or discreetly slowed development [8]–[10],[13]. The clinical course is characterized by regression of acquired skills, followed by the onset of motor, sensory, and cognitive neurological signs of increasing severity. Death usually occurs in the first decade of life [8]–[10],[13] (Table 1).

**Table 1.** Key distinctions in phenotypic presentation and age of onset between classical infantile neuroaxonal dystrophy (INAD) and atypical neuroaxonal dystrophy (NAD), both representing clinical variants within the spectrum of *PLA2G6*-related neurodegeneration.

Feature	Classic INAD	Atypical NAD
Alternate name	Infantile PLAN, typical INAD, Seitelberger's disease, infantile-onset PLAN [1]	Atypical NAD, juvenile PLAN, atypical infantile neuroaxonal dystrophy (aNAD) [1],[7]
Age of onset	Generally, in the first 2 years of life; often between 6 months and 3 years; typically around 12 months. Average onset around 15 months. Ranges 12 to 22 months [1],[7]	Presentation in early childhood, outside the infantile period. Ranges from 3 years to late adolescence [1],[3],[31]
Progression	Rapid and progressive [1],[7],[28]	Slower initial course More variable presentations [1],[7],[28]
Key motor symptoms	Psychomotor/developmental regression; hypotonia (initial, evolves to pasticity/spastic tetraparesis); cerebellar ataxia; extrapyramidal signs; loss of gross motor milestones (earliest sign); loss of fine motor milestones (progresses); loss of bulbar function (progresses); appendicular spastic hypertonia, axial hypotonia, hyperreflexia; weakness of distal muscles; diminished patellar tendon reflex; cannot stand alone; progressive loss of walking ability; poor response to calling; difficult swallowing/swallowing regression [1],[3],[7],[28],[37]	Neurodevelopmental regression; gait instability; speech delay; dystonia (common); variable combinations of ataxia and extrapyramidal features; bradykinesia; choreiform movements. Adult-onset PLAN: Parkinsonism and/or dystonia; mental deterioration [1],[3],[7],[28],[31],[37]
Visual/cognitive symptoms	Bilateral optic atrophy; nystagmus; strabismus; cognitive decline/dementia; temporo-frontal dysfunction (late loss); intellectual disability; impaired vision; global developmental delay; neurodevelopmental impairment [7],[28],[44]	Optic atrophy (less common than in classic INAD); autistic features; speech delay; mental deterioration [7],[28],[44],[57]
Neuroimaging	Cerebellar atrophy (consistent finding); mild cerebellar atrophy; cerebellar cortex hyperintensity (variable/late); globus pallidus iron deposition (variable/late/occasional); hypointensity in pallida/substantia nigra; may be absent in early stages; clava hypertrophy (useful early sign); mild cerebral hemispheres atrophy; brainstem involvement/corpus callosum hypoplasia/thinning; posterior periventricular white matter signal changes; widened bilateral cerebral hemispheres/cerebellar sulci [7],[8],[44]–[46]	Cerebellar atrophy; globus pallidus hypointensity (iron) [1],[7],[8],[28],[44]–[46]
Pathological findings	Axonal spheroids (key pathological finding, although the diagnosis is now genetic); axonal swellings and spheroid bodies in presynaptic terminals in CNS/PNS; detectable in skin, muscle, nerve, or conjunctival biopsy; peripheral nerve biopsies were long considered a gold standard [2],[3],[33],[36]	Axonal spheroids; pathological findings similar to other NBIA forms; alpha-synuclein positive Lewy bodies; dystrophic neurites; neurofibrillary tangles (in one adult atypical case) [2],[3],[7],[33],[36],[47]
Prognosis	Death generally before 10 years (average age of death 9.4 years); ranges from 6.5–14 years; relentlessly progressive, poor prognosis. No observed case of recovery of a milestone once lost; shortest life span among PLAN phenotypes; patients often succumb to respiratory decline [7],[28]	Greater survival compared to classic INAD; more variable presentation [7],[28]
Other common features	Seizures; EEG abnormalities; gastrointestinal disease; skeletal deformities; hearing loss/auditory neuropathy; elevated serum AST and LDH; sensorimotor axonal neuropathy with denervation; mitochondrial dysfunction (reduced Ca^2+^ retention capacity, decreased Ca^2+^ uptake rate, altered glutamate-evoked Ca^2+^ signals); autonomic dysfunction (temperature dysregulation, hypertension); elevated ALP/CK-MB, reduced creatinine [7],[18],[48]	More heterogeneous phenotypes; oculogyric crisis; impulse control behavior and psychosis (in juvenile Parkinson's) [7],[18],[28],[48]

### 7.1. Delay and regression of psychomotor development

The most frequent initial sign is a delay in gross motor development, especially in cephalic control, turning, and sitting [1],[2]. In many cases, children reach initial developmental milestones but subsequently experience global regression, with loss of previously acquired motor, language, and social skills [1],[3],[4],[6],[8]–[10],[13]. This regression is often the first reason for neurological consultation. Loss of balance (e.g., staggering or ataxic gait) is also commonly reported as the first affected milestone [3]. Regression initially affects gross motor skills and speech, followed by impairment of fine motor skills and bulbar function. Frontotemporal function tends to be lost at later stages [1].

### 7.2. Axial hypotonia and spasticity

Initially, generalized axial hypotonia predominates, with poor postural control. Over time, progressive lower limb–predominant spasticity appears, with pyramidal signs such as hyperreflexia, clonus, and Babinski reflex. This mixed pattern (hypotonia followed by spasticity) is characteristic of INAD [1],[3],[4],[6],[8]–[10],[13]. In some cases, hyperreflexia may be observed initially, evolving to hyporeflexia or areflexia [1],[7]. Other common findings are appendicular spastic hypertonia and the development of joint contractures [1].

### 7.3. Other manifestations

Other clinical manifestations that may appear in INAD include visual problems, including nystagmus that may be pendular or, in some cases, downbeat [1],[3],[4],[6],[8]–[10],[13], strabismus, and optic atrophy. Amblyopia and anisocoria have also been described. Cognitive problems, including progressive cognitive impairment and dementia, also occur [3],[4],[10].

Bulbar dysfunction, such as dysphagia, choking, or drooling, may progress to the need for a crushed diet or nasogastric tube feeding or gastrostomy [1]. Bulbar dysfunction is also a common cause of respiratory complications and death [1],[3],[20],[45]. Epileptic seizures can appear in early or late stages of the disease [1],[3],[20]. Other neurological signs are ataxia, tremors, dystonia, tongue twitching, dysdiadochokinesia, and Gower's sign [1],[3],[4],[6],[8]–[10],[13].

Gastrointestinal problems [1],[3], skeletal deformities such as kyphosis and scoliosis, and signs of autonomic nervous system involvement such as constipation, urinary retention or incontinence, reduced tear production, and temperature dysregulation may also appear [3]. Hearing loss or auditory neuropathy has also been described [1],[3],[4],[6],[8]–[10],[13].

## 8. Diagnosis

The diagnosis of INAD represents a clinical challenge, especially in the early stages, due to the nonspecific presentation of signs and symptoms, which may coincide with other neurological developmental pathologies. Diagnostic suspicion usually arises from the observation of progressive psychomotor delay, particularly when regression of previously acquired skills is evident, together with neurological signs such as central hypotonia, incipient spasticity, or loss of eye contact. The age of onset of classic INAD symptoms is typically between 6 months and 2 years, although there may be an initial asymptomatic period. Early signs may include slowing in the acquisition of developmental milestones or regression of milestones, as well as truncal hypotonia, strabismus, and nystagmus [7],[8],[21],[50].

The diagnosis of INAD requires a comprehensive and sequential evaluation, combining a detailed clinical evaluation with complementary neuroimaging tests and genetic studies. Brain MRI is a fundamental tool in the diagnosis of INAD, as it can reveal characteristic signs even before molecular confirmation [51].

The most relevant neuroimaging findings include early cerebellar atrophy, which may particularly affect the vermis. However, MRI may be normal in the early stages of the disease, so INAD should not be ruled out based solely on initial normal findings. Follow-up studies should be considered [8]. Bilateral hypointense signal in the globus pallidus on T2 and SWI sequences also appears, suggesting iron accumulation. This iron accumulation may be more appreciable in later stages of the disease. In some cases, hypointensity has been described in the substantia nigra and dentate nuclei. The presence of isointensity in T1 separates iron accumulation from other minerals [3],[8]. Presence of a central hypointense linear image in the globus pallidus, known as “inverse tiger eye”, is less frequent in the infantile form, but pathognomonic of other NBIA also appears.

Progressive white matter involvement without frank demyelination has been described in advanced stages [8]. Periventricular hyperintensities may also be observed [8]. Hypertrophy of the clavula (gracile tubercle formed by the nucleus and the gracile fascicle) in the medulla oblongata may be a useful finding in the early identification of INAD [6],[8].

At the neurophysiological level, INAD is characterized by EEG and may show a nonspecific pattern of generalized slowing and, in some cases, evidence of epileptiform activity [6],[7],[14].

Electromyography (EMG) and evoked potentials (EPs) can be useful to detect associated peripheral neuropathies or visual conduction disturbances. Visual evoked potentials (VEPs) can show early alterations [6],[14].

Genetic diagnosis allows confirmation of the diagnosis of INAD. It is performed by identifying bi-allelic pathogenic variants in the *PLA2G6* gene [46],[52]. Molecular genetic testing has largely replaced previously used invasive biopsies [3]. The identification of mutations in *PLA2G6* allows, in addition to definitive diagnosis, carrier detection and prenatal diagnosis [53].

The availability of gene sequencing panels associated with childhood neurodegenerative diseases, as well as whole-exome sequencing, has significantly improved diagnostic yield [2],[3],[10],[17],[20]. In selected cases, functional analyses in fibroblasts or studies in patient-derived cellular models can be used, although these approaches are more common in research settings. In this regard, an iPSC line has been generated from fibroblasts from a patient with compound heterozygous mutations in the *PLA2G6* gene. This iPSC line could be useful for studying the pathogenic mechanisms underlying INAD [12],[54]–[57]. Studies in iPSC-derived cellular models have shown phenotypes consistent with pathology in vivo, such as synaptic alterations and lysosomal dysfunction, offering a promising avenue for diagnosis and evaluation of therapies.

## 9. Treatment

To date, INAD remains a disease with no approved curative treatment [1], so management is mainly focused on palliative care and symptom control. The complexity of the pathophysiology, the rarity of the disease, and the variability in clinical presentation hinder the development of effective clinical trials [1],[4],[6].

The clinical management of INAD requires a multidisciplinary approach [1] (Table 2). Symptomatic treatment by physiotherapy is essential from the early stages to maintain joint mobility and prevent contractures [1]. Similarly, respiratory support becomes increasingly important as INAD progresses [1]–[3],[7],[20]. Truncal hypotonia and difficulties with effective coughing contribute to the risk of recurrent respiratory infections [1]–[3],[7],[20]. The leading cause of death in some studies of INAD is respiratory, probably secondary to bulbar dysfunction [1],[3],[20]. Proactive strategies are required and may include respiratory physiotherapy, the use of cough assistance devices, and, in selected cases, home noninvasive ventilation [1]. Occupational therapy is also useful to stimulate residual abilities and adapt the environment to the patient's needs. Speech therapy also helps to address speech and swallowing difficulties and to anticipate future communication needs [1],[2],[7]. From a pharmacological point of view, baclofen, diazepam, or trihexyphenidyl are used for spasticity and dystonia, although their efficacy may be limited and require careful monitoring for side effects [3],[7],[15],[21]. In cases of severe dystonia, botulinum toxin and intrathecal administration of baclofen by implantable pump in specialized centers have been explored. Epileptic seizures, present in some patients, are treated with standard anticonvulsant drugs, adapted to each case. An example is the use of Levetiracetam [3]. Other drugs can also be used to treat specific symptoms, such as amlodipine for hypertension or pantoprazole for gastrointestinal problems [3]. Measures should be taken to prevent secondary complications such as respiratory infections (frequent due to bulbar involvement) and pressure ulcers. In cases of feeding difficulties, placement of a percutaneous endoscopic gastrostomy tube may be necessary. In cases of severe respiratory complications, a tracheostomy may be required [3].

**Table 2.** Experimental therapies and research strategies for *PLA2G6*-associated neurodegeneration (PLAN).

Experimental therapy / Research strategy	Proposed objective / Current status
RT001 (di-deuterated ethyl ester of linoleic acid)	Evaluate its efficacy in patients with INAD. It is investigated for its potential protective effect against lipid peroxidation. Clinical studies with RT001 (open-label study) are currently underway. An initial two-case study suggested possible signals of efficacy, but the need for larger controlled studies is recognized [5],[24].
Gene therapy / Gene replacement	Replace the function of the defective *PLA2G6* gene by inserting a correct version of the gene, potentially using viral vectors. Considered as a potential future approach for treatment [2].
Targeting Simultaneous Oxidation and Iron Chelation	Address lipid peroxidation and iron accumulation in the brain simultaneously. It is a strategy being considered in research [2].
Semaglutide (GLP1 receptor agonist)	Reduce neuronal loss and improve symptoms. Showed promising results in an INAD mouse model, decreasing neuronal loss and improving symptoms. Requires much more research; not currently recommended for PLAN in humans due to risks and lack of clinical data in this population [2],[24].
Ambroxol, azoraminde, genistein	Decrease cellular abnormalities observed in preclinical models (such as Drosophila flies and patient-derived cells lacking *PLA2G6*). Identified as possible candidates in early stages of preclinical research [24].
Desipramine	Decrease cellular abnormalities in preclinical models (flies and cells without PLA2G6). However, not recommended for the treatment of PLAN in humans due to potential risks and lack of data supporting its clinical benefit in patients [24].
Iron chelating agents (such as Deferiprone)	Reduce cerebral iron accumulation, which is observed on neuroimaging in some PLAN patients. They have been evaluated in PLAN patients, showing variable effects on cerebral iron on MRI but without evidence of clinical benefit. Not recommended for PLAN due to risks and lack of demonstrated clinical benefit. It was already tested in a randomized controlled trial for another NBIA [24].
Patient-derived cellular models (Induced pluripotent stem cells - iPSC)	Essential tool for research, used to study the pathogenic mechanisms of the disease and to evaluate new therapies in vitro. Not a direct therapy for the patient, but a fundamental platform for drug discovery and testing. (Note: This is a research tool for developing therapies, not a therapy administered directly to patients) [12],[55],[56],[57].

Given the progressive and severe nature of the disease, it is also important to offer psychological and social support to both patients and their families [1],[43].

Therapeutic prospects under development focus primarily on correcting the underlying molecular defect through gene therapy [2]. Gene replacement therapy seeks to insert a functional copy of the *PLA2G6* gene using viral vectors, such as adeno-associated virus. The human *PLA2G6* gene, with a coding sequence of just over 2.4 kb, is of adequate size to be packaged into a viral vector [2]. Different approaches for the delivery of these vectors are being considered, including the intravascular or intracerebrospinal route to directly target the CNS. However, the multiplicity of mutations in the *PLA2G6* gene presents a major challenge [2].

Another therapeutic strategy under development is precision gene editing, using tools such as CRISPR/Cas9. This technology has the potential to directly correct deleterious mutations in the DNA of affected cells. With the large number of missense mutations observed in the *PLA2G6* gene, gene or base correction is considered for therapy [2].

Also, enzyme replacement therapy is another potential strategy explored for rare enzyme-defective diseases such as INAD. However, there are specific procedural challenges for this therapy in the brain, including the need to reach mitochondria. Preclinical studies in animal models are in progress [2].

In addition to addressing the primary genetic defect, approaches that seek to modulate common pathological pathways in INAD are being investigated. This includes strategies to reduce lipid peroxidation using compounds such as deuterated polyunsaturated fatty acids (D-PUFAs) and antioxidants [2],[5]. Combination therapies that could simultaneously target oxidation and iron chelation are also being considered [2].

One of the most studied approaches is the use of the D-PUFA RT001, a deuterated form of linoleic acid designed to protect lipids from oxidative damage [5],[38]. Deuteration at specific points in the molecule slows peroxidation and protects against the formation of toxic by-products [5]. This treatment has been evaluated in experimental models, shown to ameliorate locomotor deficits in flies lacking the *PLA2G6* orthologue and to reverse mitochondrial abnormalities in fibroblasts derived from patients with pathogenic mutations in *PLA2G6*. D-PUFAs are the only targeted therapy shown to ameliorate mitochondrial pathology in a *PLA2G6*-deficient disease model. RT001 was also evaluated in two subjects with INAD, aged 34 and 10 months [5]. Treatment was well tolerated, with no drug-related adverse events, and plasma levels of deuterated fatty acids reached and maintained the intended target. There was limited slowing of disease progression but promoted improvements in alertness, participation, vocalization, and fine motor control in one of the subjects during the first few months, although a decline was reported later [5].

Other conventional antioxidants, such as vitamin E, have also been considered due to their potential role in lipid peroxidation pathways [40]. Vitamin E helped in cellular models of PLAN in preclinical studies. In one particular case, an affected patient with INAD was treated with a supplement containing DHA and EPA, along with vitamin E. However, conventional antioxidants have not been tested in controlled trials for PLAN, and further studies are required to determine whether vitamin E is safe and effective in animal models and humans, considering the risks associated with vitamin E excess [40].

Antioxidants may require direct targeting of mitochondria [29],[30]. Mitochondria-targeted antioxidants, such as MitoQ, MitoVitE, and MitoApocynin, have been shown to protect against mitochondrial lipid peroxidation in in vitro and in vivo studies. MitoQ, in particular, has been safely administered orally in human studies for other conditions and has been shown to reduce mitochondrial oxidative damage [30].

In addition, the use of agents with antioxidant and iron-chelating properties has been investigated in in vitro studies [24]. It is considered that combination therapies, simultaneously addressing oxidation and iron chelation, might be more effective. However, iron chelation with deferiprone in PLAN patients is not currently recommended because, although stable or decreased brain iron levels have been observed, there has been a worsening of clinical symptoms and no evidence of clinical benefit [24].

Finally, other compounds such as ambroxol, azoraminde, desipramine, and genistein have been used in cellular models derived from patients lacking *PLA2G6* [24]. These drugs decreased the abnormalities observed in flies and cells, but additional validation in animal models and evidence of safety and efficacy are needed before they can be considered for human testing. Specifically, desipramine is not recommended for PLAN [24].

A relevant aspect is the possible connection between lipid peroxidation and the accumulation of iron and lipofuscin [58]. Iron ions in their reduced state (Fe^2+^) play a crucial role in catalyzing lipid peroxidation through the Fenton reaction, which generates lipid-damaging reactive oxygen species (ROS) [58]. In fact, Fe^2+^-catalyzed non-enzymatic lipid peroxidation is considered a critical event in ferroptosis, a type of iron-dependent programmed cell death. The addition of exogenous iron, such as ferric ammonium citrate, has been shown to induce ROS production and lipid peroxidation specifically in mitochondria, which is critical for ferroptosis induced by this iron [58]. Lipid peroxidation is not only linked to cell death but also to lipofuscin accumulation. Lipofuscin is a complex material formed from products of lipid peroxidation and oxidized cross-linked proteins [58]. Lipid peroxidation occurring in mitochondria in an iron-dependent manner can directly provide material for the formation of this compound. Once formed, lipofuscin can even bind redox-active iron, creating a cycle by catalyzing the generation of more ROS in senescent cells. Oxidative stress, along with other factors such as inflammation, alters cellular iron homeostasis, leading to iron overload and damage [58]. This suggests a feedback loop where oxidative damage, including lipid peroxidation, may exacerbate iron accumulation in mitochondria.

Models based on cells derived directly from patients, such as fibroblasts, and more complex cellular models derived from induced pluripotent stem cells (iPSCs), which can differentiate into neurons, have been particularly valuable for in vitro research [2],[12],[59].

A study using fibroblasts obtained from a skin biopsy of a subject affected by INAD at the age of 25 years, carrying the homozygous c.316C > T, p.Arg106Cys variant in *PLA2G6*, characterized cellular abnormalities such as a significant reduction in mitochondrial elongation and an increase in circularity in patient fibroblasts, indicating that the filamentous connection of mitochondria was altered in favor of a fragmented network [39]. Mitochondrial functionality was analyzed by measuring oxygen consumption rate (OCR) and extracellular acidification rate (ECAR) using microscale oxygraphy (Seahorse assay) [39]. Although no significant differences in basal OCR were found, the OCR/ECAR ratio was significantly decreased in mutant cells, suggesting metabolic imbalance. A glycolysis assay showed only a slight increase in basal glycolytic parameters, but a significant increase in glycolysis under conditions of cellular stress and increased energy demand in patient fibroblasts [24],[39].

More complex in vitro models have been developed using induced pluripotent stem cells (iPSCs), which have the advantage of being able to be differentiated into disease-relevant cell types such as neurons. Human iPSC lines (hiPSCs) carrying specific mutations in *PLA2G6* have enabled the evaluation of new therapies [12]. The hiPSC line ONHi001-A has been generated from fibroblasts of a 6-year-old patient with INAD carrying the mutations c.517C > T (p.Q173X) and c.1634A > G (p.K545R) [12]. Non-integrable episomal vectors were used for reprogramming, and the line was maintained by a feeder cell-free culture method. The ONHi001-A line was confirmed to show typical human iPSC/embryonic stem cell morphology and express pluripotency markers such as OCT4 [12]. Studies in iPSC-derived cell models have shown phenotypes consistent with in vivo pathology, such as synaptic alterations and lysosomal malfunction [2],[12],[59]. However, none of these therapies are currently approved for clinical use.

## 10. Conclusions

INAD is a rare neurodegenerative disease characterized by progressive motor and cognitive impairment, with diagnosis based on a combination of clinical evaluation, neuroimaging, and genetic confirmation. Although early clinical diagnosis remains a challenge, advances in molecular genetics and neuroimaging have enabled more accurate and rapid diagnosis, facilitating therapeutic strategies. Management of INAD focuses primarily on symptomatic treatment and palliative support, with a multidisciplinary approach encompassing physiotherapy, speech therapy, spasticity management, seizure control, and nutritional and respiratory support. There are no treatments for the disease that can slow its progression or improve quality of life, with life expectancy being reduced, especially in early-onset cases. Approaches such as the use of adeno-associated viral vectors (AAV) to restore normal function in affected cells of the central nervous system or gene editing using CRISPR/Cas9 are being investigated. Continued research into the molecular mechanisms underlying the pathogenesis of INAD will identify potential therapeutic targets and advance the development of treatments.

## 11. Limitations of current literature and future challenges

The understanding and management of INAD and PLAN present inherent challenges, largely stemming from their extreme rarity. This low prevalence directly impacts the availability and level of evidence in scientific literature. Due to this fragmentation and the lack of large-scale studies, most of the available knowledge comes from individual case reports and small case series. There is a notable absence of large-scale controlled studies, which limits the strength of conclusions and makes it difficult to obtain high-level evidence to evaluate the efficacy of interventions. As a result, many management recommendations are predominantly based on clinical expert consensus, as reflected in clinical management guidelines. Although international cohorts and patient registries are being formed, studies with significant numbers of patients remain limited.

## Use of AI tools declaration

The authors declare they have not used Artificial Intelligence (AI) tools in the creation of this article.
